# Early prediction of cognitive impairment in adults aged 20 years and older using machine learning and biomarkers of heavy metal exposure

**DOI:** 10.1016/j.crtox.2024.100198

**Published:** 2024-10-18

**Authors:** Ali Nabavi, Farimah Safari, Mohammad Kashkooli, Sara Sadat Nabavizadeh, Hossein Molavi Vardanjani

**Affiliations:** a Student Research Committee, Shiraz University of Medical Sciences, Shiraz, Iran; b Department of Otolaryngology, Otolaryngology Research Center, Shiraz University of Medical Sciences, Shiraz, Iran; c Research Center for Traditional Medicine and History of Medicine, Shiraz University of Medical Sciences, Shiraz, Iran

**Keywords:** Cognitive impairment, Heavy metal exposure, Predictive models

## Abstract

**Background:**

Cognitive impairment poses a growing health challenge as populations age. Heavy metals are implicated as environmental risk factors, but their role is not fully understood. Machine learning can integrate multi-factorial data to predict cognitive outcomes.

**Objective:**

To develop and validate machine learning models for early prediction of cognitive impairment risk using demographics, clinical factors, and biomarkers of heavy metal exposure.

**Method:**

A retrospective analysis was conducted using 2011–2014 NHANES data. Participants aged ≥ 20 underwent cognitive testing. Variables included demographics, medical history, lifestyle factors, and blood and urine levels of lead, cadmium, manganese, and other metals. Machine learning algorithms were trained on 90 % of data and evaluated on 10 %. Performance was assessed using metrics like accuracy, AUC, and sensitivity.

**Result:**

A final sample of 2,933 participants was analyzed. The stacking ensemble model achieved the best performance with an AUC of 0.778 for test data, sensitivity of 0.879. Important predictors included age, gender, hypertension, education, urinary cadmium and blood manganese levels.

**Conclusion:**

Machine learning can effectively predict cognitive impairment risk using comprehensive clinical and exposure data. Incorporating heavy metal biomarkers enhanced prediction and provided insights into environmental contributions to cognitive decline. Prospective studies are needed to validate models over time.

## Highlights

• Developed ML models to predict cognitive impairment risk using demographics, clinical factors, and heavy metal biomarkers from NHANES data.
• Stacking ensemble model achieved best performance (AUC 0.778) on test data, effectively predicting risk with comprehensive variables.
• Urinary cadmium and blood manganese levels enhanced prediction, offering insights into environmental factors in cognitive decline risk.

## Graphical abstract

**Figure:**
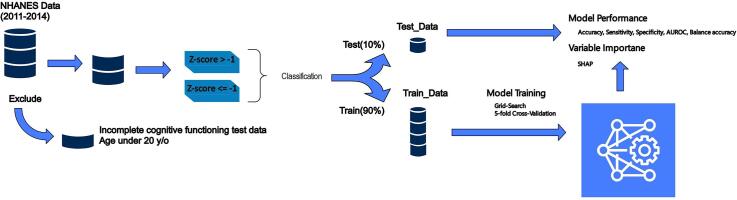

## Introduction

The rising prevalence of cognitive dysfunction presents a significant challenge to modern healthcare systems worldwide. As the global population ages, conditions characterized by cognitive impairment, such as dementia, are expected to impact an ever-increasing number of individuals (Crimmins et al., 2011, Ngandu et al., 2015). Cognitive dysfunction manifests in various forms, ranging from mild memory deficits to severe impairments that profoundly disrupt essential functions like communication, behavior, decision-making, and overall quality of life (Organization, 2004, Opara, 2012). Alarmingly, nearly half of those experiencing mild cognitive impairment transition to dementia within three years, highlighting the imperative need for early intervention and robust support systems to mitigate the mounting burden on individuals, caregivers, and society. Proactive measures to identify and address the underlying risk factors contributing to cognitive decline are crucial in combating this escalating public health crisis (Pal et al., 2018).

Identifying the underlying factors contributing to cognitive impairment has garnered significant attention in recent years. Heavy metals (HMs) have emerged as a crucial environmental factor implicated in cognitive dysfunction among both children and adults (Althomali et al., 2024). Lead, mercury, arsenic, and cadmium are heavy metals believed to impair cognitive functions through various neurotoxic mechanisms in the brain. These toxic elements can breach the blood–brain barrier, accumulating in brain tissues and disrupting crucial neurotransmitter systems responsible for memory formation, executive function, social cognition, and motor coordination (Althomali et al., 2024, Ouyang et al., 2023, Nguyen and Kim, 2022). The neurotoxic mechanisms of heavy metals are multifaceted, including interference with calcium signaling pathways, induction of oxidative stress, and impairment of mitochondrial function – all of which can ultimately lead to neuronal injury and cognitive deficits. Several studies have shown a strong correlation between high levels of heavy metals in the blood and reduced cognitive function, indicating the potential use of blood biomarkers in predicting and tracking cognitive impairment.(Ouyang et al., 2023, Nguyen and Kim, 2022, Karri et al., 2016, Nguyen and Kim, 2023).

The advent of machine learning (ML) techniques has opened up promising avenues for screening cognitive impairment related to heavy metal exposure. By leveraging the power of ML algorithms, researchers can integrate multiple risk factors, such as demographics, environmental exposures, and biomarkers, into predictive modeling tools. This is enabled through various ML techniques' ability to process huge and complex datasets and perform predictive analytics. Particularly, ML methods can surpass traditional statistical approaches by effectively managing extensive and complex nonlinear data without depending on preexisting assumptions, and by capturing intricate connections among predictors (Liang et al., 2022). Furthermore, ML methods possess the advantage of having simple performance metrics like area under curve (AUC) and accuracy to objectively compare different models (Bradley, 1997).

As ML models transition from research to clinical use, focusing on increasing the quality and quantity of data through iterative data collection, preparation, and testing can help address challenges like ensuring reliability for deployment. The data-centric ML approach combined with algorithm refinement may achieve the best outcomes for this proposed research to develop predictive models for CI (Liang et al., 2022).

This study aims to develop and validate an optimal machine learning model for the early prediction of cognitive impairment risk in older adults. By evaluating multiple models on a diverse set of variables, including demographics, main risk factors, and heavy metal exposure levels, we attempt to identify the approach with the highest predictive performance. The resulting model could enable early risk stratification and interventions to combat cognitive decline.

## Method

### Study design and data Source

This research involved a retrospective analysis of the National Health and Nutrition Examination Survey (NHANES) data spanning from 2011 to 2014. NHANES is an extensive cross-sectional survey orchestrated by the National Center for Health Statistics that aims to gauge the health and nutritional conditions of American adults and children. It employs a multistage probability sampling technique, ensuring that the samples reflect the U.S. population adequately (Taylor et al., 2020). This survey amalgamates interview data with physical examinations and laboratory tests, providing a comprehensive dataset for health-related research (Casagrande et al., 2021).

From an initial 19,931 NHANES participants for the 2011–2014 cycles, we applied exclusion criteria to obtain our final analytic sample for predicting cognitive impairment risk. Individuals under 20 years of age and those with incomplete cognitive functioning test data were excluded, leaving 2,933 eligible participants.

### Data collection

Data for the selected variables were collected from the National Health and Nutrition Examination Survey (NHANES) dataset. Demographic information and lifestyle factors were gathered through questionnaires and interviews conducted as part of the NHANES protocol.

For biomarkers indicative of heavy metal exposure, we utilized data from the biological specimen component of NHANES, ensuring accurate and reliable measures of environmental exposures. These biomarkers, quantified in blood and urine, included a comprehensive set of substances such as inorganic and ethyl mercury, lead, cadmium, selenium, manganese, various arsenic compounds, and additional metals like barium, cobalt, cesium, molybdenum, antimony, tin, thallium, tungsten, and uranium.

We categorized several variables to facilitate analysis. Smoking status was classified as never smoker (smoked < 100 cigarettes lifetime), past smoker (smoked ≥ 100 cigarettes lifetime but not currently), or current smoker (smoked ≥ 100 cigarettes lifetime and currently smokes daily/sometimes). Alcohol consumption was categorized as non-drinker (<12 drinks lifetime and past year), former drinker (≥12 drinks lifetime but not past year), or current drinker (≥12 drinks in past year, with average weekly intake collected). Family income-to-poverty ratios were grouped as < 1.30, 1.30–3.49, or ≥ 3.50 based on reported family income and poverty thresholds.

Depression severity was assessed using the Patient Health Questionnaire (PHQ), a 9-item screening tool where each item is rated on a 0–3 scale, with a maximum total score of 27 indicating increasing depression severity.

### Variable selection

The process of selecting variables for our analysis was rigorous and multi-staged, combining literature review, domain expertise, and data-driven approaches to evaluate their predictive power for cognitive impairment. Initially, we conducted an extensive review of literature on cognitive impairment, environmental toxins, and health outcomes to identify potential predictors. This review led to the consideration of a wide range of variables including demographic characteristics, lifestyle and health factors, and biomarkers indicative of heavy metal exposure.

Following the literature review, we refined our variable list based on established or suspected associations with cognitive function. We included demographic characteristics such as age, gender, race/ethnicity, education level, marital status, and family poverty-to-income ratio (PIR). Lifestyle and health factors encompassed physical activity (including minutes of vigorous and moderate work-related activity, minutes spent walking or biking for transport, and minutes engaged in moderate recreational activities), smoking status, alcohol consumption, hypertension, health insurance coverage, and depression severity.

To further refine our selection, we conducted a missing data analysis as detailed in the Preprocessing section. Variables with more than 20 % missing data were excluded to ensure the reliability of our findings. Subsequently, we employed Recursive Feature Elimination (RFE) in conjunction with a Random Forest estimator. This data-driven approach complemented our initial literature-based selection, allowing us to identify and focus on the most significant predictors of cognitive impairment.

### Cognitive performance evaluation

In this study, cognitive capabilities were evaluated using a sequence of established neuropsychological tests designed to capture various cognitive domains. The assessment protocol commenced with the Consortium to Establish a Registry for Alzheimer's Disease Word Learning (CERAD-WL) test, proceeded with the Animal Fluency (AF) test, advanced to the Digit Symbol Substitution Test (DSST), and culminated with the CERAD Delayed Recall (CERAD-DR) test. The CERAD-WL test involves multiple attempts at word recall to assess learning and memory, culminating in a final delayed-recall phase scored out of 10 points (Rosen, 1983). The initial learning phase permits a maximum score of 30. The AF test challenges participants to generate as many animal names as possible within a minute, testing their verbal fluency and executive functioning, with each correct response earning a point (Clark et al., 2009). The DSST, testing for cognitive processing speed and working memory, asks participants to match symbols with numbers within two minutes, with each correct pairing contributing to a possible total of 133 points (Brody et al., 2019.).

**Fig. 1:**
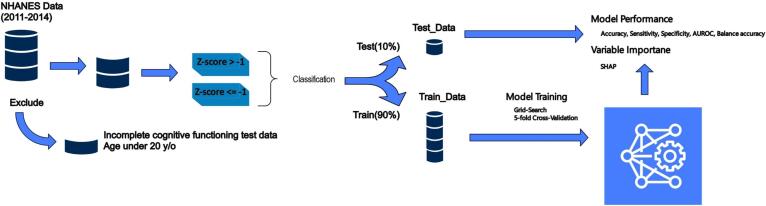
Proposed methodology for developing machine learning models to predict cognitive impairment risk. NHANES: National Health and Nutrition Examination Survey, y/o: years old, AUCROC: Area under the Receiver Operating Characteristic Curve.

To quantify overall cognitive performance, we calculated a composite Z-score for each participant. First, individual Z-scores were computed for each test (CERAD-WL, AF, DSST, and CERAD-DR) using the formula Z = (x − µ) / σ, where x is the participant's test score, µ is the population mean, and σ is the standard deviation. The composite Z-score was then derived by taking the arithmetic mean of these four individual Z-scores. This method provides a standardized measure of cognitive performance across all assessed domains. In line with current neuropsychological standards, we defined potential cognitive impairment as a composite Z-score below −1. (Fig. 1) (Frith et al., 2018, Wirth et al., 2017).

### Preprocessing

Before initiating model development, we performed several preprocessing steps to refine the NHANES dataset for enhanced analytical accuracy. Responses that were indicated as 'refusal' or marked as 'unknown' were treated as missing values for the following variables: Diabetes, Gender, Age, Race/Ethnicity, Education Level, Marital Status, Family PIR (Poverty Income Ratio), Smoking, Hypertension, and Alcohol. We set a threshold to exclude variables and participants with significant gaps in data. Specifically, variables with more than 20 % missing data and participants lacking essential information were excluded to ensure the reliability of our findings.

Categorical and ordinal variables were transformed through one-hot encoding, converting them into a numerical format compatible with machine learning algorithms. This step was crucial for seamlessly integrating these features into the modeling process. We then applied scalar normalization methods for numerical variables to promote uniform contributions across all features and to prepare the data for subsequent outlier detection.

Following normalization, we addressed outliers in the dataset using the DBSCAN (Density-Based Spatial Clustering of Applications with Noise) technique, a robust outlier detection method. DBSCAN operates as a clustering algorithm that identifies outliers by estimating the density of data points in the normalized space, effectively segregating instances that do not conform to typical data densities.

For DBSCAN implementation, we used a composite distance metric to handle both numerical and categorical variables simultaneously. For numerical variables, we employed the Euclidean distance, which is effective for normalized continuous data. For categorical variables (which were one-hot encoded), we used the Hamming distance, which measures the proportion of differing attributes between two samples. These distance measures were combined using a weighted sum approach, with weights proportional to the number of features of each type. This composite metric allows DBSCAN to consider both numerical and categorical features in its density calculations.

To manage missing data within the dataset, we adopted a tailored imputation strategy. For categorical variables, we imputed missing values using the mode, while for numerical variables, we used the mean. This method ensured the preservation of the dataset's integrity and allowed for the comprehensive inclusion of all pertinent cases in the subsequent analysis.

To counteract the dataset's significant imbalance, particularly its skew towards individuals without hearing impairment, we employed the Synthetic Minority Over-sampling Technique (SMOTE). This technique improves model efficacy on imbalanced datasets by generating synthetic instances of the minority class. By doing so, SMOTE balances the dataset distribution, fostering a more equitable environment for model training and enhancing the overall robustness of our predictive analysis.

We implemented Recursive Feature Elimination (RFE) in tandem with a Random Forest estimator for the feature selection process. RFE methodically removes features deemed least important by the estimator, based on its rankings of feature importance. This iterative elimination continues until the most significant predictors for hearing impairment are isolated, effectively streamlining the set of variables used in the final model. This strategy improved the model's performance and interpretability by focusing on the most impactful predictors.

### Machine learning model development and evaluation

To build an optimal predictive model for cognitive impairment risk, we performed a comprehensive evaluation of several state-of-the-art machine learning algorithms. Initially, we partitioned the dataset into a pre-training set (90 %) and a test set (10 %), with the latter reserved exclusively for final external validation. We then conducted a 5-fold cross-validation experiment on the pre-training set, further dividing each fold into training (80 %) and validation (20 %) subsets. This stratified approach ensured a thorough assessment of model stability and generalizability.

Our preprocessing pipeline, applied independently to each fold, encompassed several critical steps. We normalized the training data and applied the resulting scaler to both validation and test sets. Variable selection was performed using Recursive Feature Elimination (RFE) on the training data, while outlier detection utilized DBSCAN, with the resulting model applied to identify outliers in validation and test sets.

We evaluated various algorithms, including Stochastic Gradient Boosting, Multilayer Perceptron (MLP), CatBoost, Support Vector Machines (SVM), and a stacked ensemble approach. For each model, we conducted hyperparameter optimization using GridSearchCV, confining this process to the training set to avoid overfitting. The optimized models were then validated using the held-out validation set within each fold.

To comprehensively assess model performance, we employed a diverse set of metrics: accuracy, sensitivity, specificity, area under the receiver operating characteristic curve (AUC-ROC), and balanced accuracy. These metrics collectively provide insights into various aspects of model capability, from correctly identifying true positives and negatives to overall predictive power and performance on imbalanced datasets. We computed these metrics for both training and validation sets across all folds, summarizing the results as means and standard deviations to capture model stability. Following the cross-validation process, we performed an external validation using the initially sequestered test set, providing an unbiased estimate of real-world model performance.

### Model interpretation

To improve the interpretability of our models, we employed SHAP (SHapley Additive exPlanations), a comprehensive framework that delivers clear explanations by attributing the contributions of individual features to the model's predictions. A key tool in our analysis was the SHAP beeswarm plot, which compiles and displays the SHAP values for all features within the dataset. This visualization highlights the extent of each feature's influence on the model’s output, with color coding to indicate whether a feature positively or negatively impacts the likelihood of hearing loss. This not only clarifies the role of each feature but also enhances our understanding of their effects.

The integration of SHAP into our analysis effectively closed the gap between model accuracy and interpretability, providing a deeper insight into the data's intrinsic patterns and relationships.

Data analysis and model development were conducted using Python (version 3.8) as the primary programming language. We utilized various Python libraries for data preprocessing, machine learning implementation, and statistical analysis. Our machine learning models were primarily built using the scikit-learn library. For data visualization, we employed matplotlib. All analyses were performed in Jupyter Notebooks to ensure reproducibility and facilitate collaborative research (Pedregosa, 2011, Hunter, 2007, Kluyver et al., 2016).

## Result

### Clinicopathological characteristics

A total of 2933 participants were enrolled according to inclusion and exclusion criteria. The baseline characteristics of the participants are presented separately for healthy and CI individuals in Table 1, Table 2. The sample comprised 1428 (48.69 %) males and 1505 (51.31 %) females. The majority were non-Hispanic white (52.84 %), followed by non-Hispanic black (26.3 %), Mexican American (9.69 %) and other Hispanic (11.17 %). Education levels varied, with the highest proportions having some college education (28.15 %) or being high school graduates (23.4 %).

**Table 1:** Comparison of continuous demographic and clinical variables between healthy and cognitive impairment groups. SD: standard deviation, BMI: body mass index, ug/L: micrograms per liter. Statistical significance is defined as p < 0.05.

**Characteristic**	**Total, mean (SD)**	**Normal group, mean (SD)**	**CI group, mean (SD)**	**P value**
**Age**	**69.48 (6.77)**	**68.92 (6.59)**	**74.38 (6.39)**	**0.000**
**BMI**	**29.06 (6.36)**	**29.22 (6.41)**	**27.61 (5.69)**	**0.000**
**Minutes vigorous-intensity work**	29.03 (242.96)	30.18 (252.17)	15.20 (64.65)	**0.066**
**Minutes moderate-intensity work**	63.17 (340.01)	59.92 (258.96)	102.23 (837.11)	**0.548**
**Minutes walk/bicycle for transportation**	30.87 (330.66)	31.39 (343.82)	24.65 (54.24)	**0.475**
**Minutes moderate recreational activities**	38.38 (58.68)	38.04 (58.60)	42.60 (59.68)	**0.380**
**Mercury, Inorganic, blood (ug/L)**	0.28 (0.23)	0.29 (0.22)	0.26 (0.22)	**0.051**
**Mercury, ethyl, blood (ug/L)**	0.11 (0.03)	0.11 (0.03)	0.11 (0.02)	**0.467**
**Mercury, total, blood (ug/L)**	**1.76 (2.51)**	**1.79 (2.53)**	**1.57 (2.38)**	**0.194**
**Mercury, urine (ug/L)**	**0.61 (1.91)**	**0.61 (1.98)**	**0.59 (1.02)**	**0.864**
**Lead, blood (ug/L)**	**1.88 (1.63)**	**1.86 (1.65)**	**2.06 (1.52)**	**0.066**
**Lead, urine (ug/L)**	**0.68 (0.95)**	**0.65 (0.85)**	**0.92 (1.59)**	**0.105**
**Cadmium, blood (ug/L)**	**0.52 (0.45)**	**0.52 (0.46)**	**0.52 (0.37)**	**0.788**
**Cadmium, urine (ug/L)**	**0.45 (0.47)**	**0.44 (0.46)**	**0.56 (0.57)**	**0.048**
**selenium, blood (ug/L)**	195.07 (33.60)	**195.87 (33.70)**	188.45 (32.02)	**0.001**
**manganese, blood (ug/L)**	9.40 (4.04)	**9.41 (3.73)**	9.25 (6.01)	**0.059**
**Manganese, urine (ug/L)**	**0.16 (0.41)**	**0.16 (0.40)**	**0.18 (0.52)**	**0.007**
**total Arsenic, urine (ug/L)**	18.64 (51.26)	18.89 (53.55)	16.31 (19.37)	**0.298**
**Arsenous acid, urine (ug/L)**	0.47 (1.96)	0.47 (2.07)	0.45 (0.36)	**0.790**
**Arsenic acid, urine (ug/L)**	0.63 (1.24)	0.64 (1.30)	0.60 (0.06)	**0.330**
**Arsenobetaine, urine (ug/L)**	10.92 (43.63)	11.27 (45.64)	7.72 (16.46)	**0.089**
**Arsenocholine, urine (ug/L)**	0.21 (0.67)	0.22 (0.70)	0.16 (0.11)	**0.019**
**Dimethylarsinic acid, urine (ug/L)**	5.74 (8.06)	5.63 (8.17)	6.71 (6.91)	**0.120**
**Monomethylarsonic acid, urine (ug/L)**	0.78 (2.23)	0.77 (2.34)	0.88 (0.68)	**0.265**
**Barium, urine (ug/L)**	**1.62 (4.05)**	**1.64 (4.23)**	**1.44 (1.64)**	**0.386**
**Cobalt, urine (ug/L)**	0.58 (1.84)	0.58 (1.92)	0.53 (0.84)	**0.622**
**Cesium, urine (ug/L)**	4.88 (3.27)	4.82 (3.22)	5.34 (3.66)	**0.195**
**Molybdenum, urine (ug/L)**	47.32 (42.22)	46.96 (42.53)	50.56 (39.36)	**0.405**
**Antimony, urine (ug/L)**	0.07 (0.18)	0.07 (0.19)	0.06 (0.05)	**0.313**
**Tin, urine (ug/L)**	1.87 (5.25)	1.68 (4.76)	3.55 (8.33)	**0.035**
**Thallium, urine (ug/L)**	0.16 (0.13)	0.16 (0.12)	0.18 (0.15)	**0.467**
**Tungsten, urine (ug/L)**	0.11 (0.25)	0.10 (0.23)	0.16 (0.38)	**0.114**
**Uranium, urine (ug/L)**	0.01 (0.03)	0.01 (0.02)	0.02 (0.07)	**0.379**

**Table 2:** Comparison of categorical demographic and clinical variables between healthy and cognitive impairment groups. N: number, CI: cognitive impairment, FMPIR: family poverty to income ratio. Statistical significance is defined as p < 0.05.

**Characteristic**		**Total, N (%)**	**Normal group, N (%)**	**CI group, N (%)**	**P value**
**Gender**	**Male**	1428 (48.69)	1257 (47.76)	171 (56.81)	**0.003**
	**Female**	1505 (51.31)	1375 (52.24)	130 (43.19)	
**Race**	**Mexican American**	257 (9.69)	249 (10.46)	8 (2.96)	**0.002**
	**Other Hispanic**	296 (11.17)	265 (11.13)	31 (11.48)	
	**Non-Hispanic White**	1401 (52.84)	1251 (52.54)	150 (55.56)	
	**Non-Hispanic Black**	697 (26.30)	616 (25.87)	81 (30.00)	
	**Other Race**	282 (9.61 %)	251 (9.54 %)	31 (10.3 %)	
**Marital Status**	**Married**	1614 (55.08)	1471 (55.95)	143 (47.50)	**0.000**
	**Widowed**	576(19.66)	472 (17.95)	104 (34.55)	
	**Divorced**	416 (14.20)	390 (14.79)	26 (8.64)	
	**Separated**	78 (2.66)	71 (2.70)	7 (2.33)	
	**Never married**	167(5.70)	153 (5.80)	14 (4.65)	
	**Living with partner**	79 (2.70)	72 (2.81)	7 (2.33)	
**Education Level**	**Less than 9th grade**	330 (11.26)	303 (11.52)	27 (8.97)	**0.001**
	**9-11th grade**	416(14.19)	374 (14.22)	42 (13.95)	
	**High school graduate**	686 (23.40)	607 (23.08)	79 (26.25)	
	**Some college**	825(28.15)	747 (28.40)	78 (25.91)	
	**College graduate or above**	674 (22.30)	599 (22.78)	75 (24.92)	
**Health insurance**	**Yes**	2690 (91.88)	2407 (91.55)	283 (94.33)	**0.092**
	**No**	239 (8.12)	222 (8.45)	17 (5.67)	
**FMPIR**	**<1.30**	821 (27.99)	711 (27.01)	89 (29.57)	**0.000**
	**1.30**–**3.49**	1145 (39.04)	1053 (40.01)	123 (40.86)	
	**>3.50**	967 (32.97)	868 (32.98)	89 (29.57)	
**Smoking, N (%)**	**Never smoker**	311(20.91)	284 (21.07)	27 (19.42)	**0.843**
	**Past smoker**	61 (4.10)	56 (4.15)	5 (3.60)	
	**Current smoker**	1115 (74.98)	1008 (74.78)	107 (76.98)	
**Drinking status**	**Non-drinker**	440 (15.00)	421 (16.00)	42 (13.95)	**0.179**
	**Former-drinker**	1027 (35.02)	894 (33.97)	108 (35.88)	
	**Current-drinker**	1466 (49.98)	1317 (50.03)	151 (50.17)	
**Diabetes**	**Yes**	688 (24.58)	609 (24.30)	79 (27.05)	**0.023**
	**No**	2111 (75.42)	1898 (75.70)	213 (72.95)	
	**Borderline**	132 (4.5 %)	123 (4.68 %)	9 (2.99 %)	
**Hypertension**	**Yes**	1829 (62.49)	1620 (61.67)	209 (69.67)	**0.022**
	**No**	1098 (37.51)	1007 (38.33)	91 (30.33)	

Almost 90 % of participants were classified as healthy, with a significantly higher proportion of younger, less depressed male adults. Regarding comorbidities, the CI group included more patients with hypertension and diabetes. For laboratory findings, the levels of urine Tin, Arsenocholine, Manganese, and Cadmium alongside the levels of blood Selenium and Lead were significantly higher in the CI group.

### The performance of ML models

**Fig. 2:**
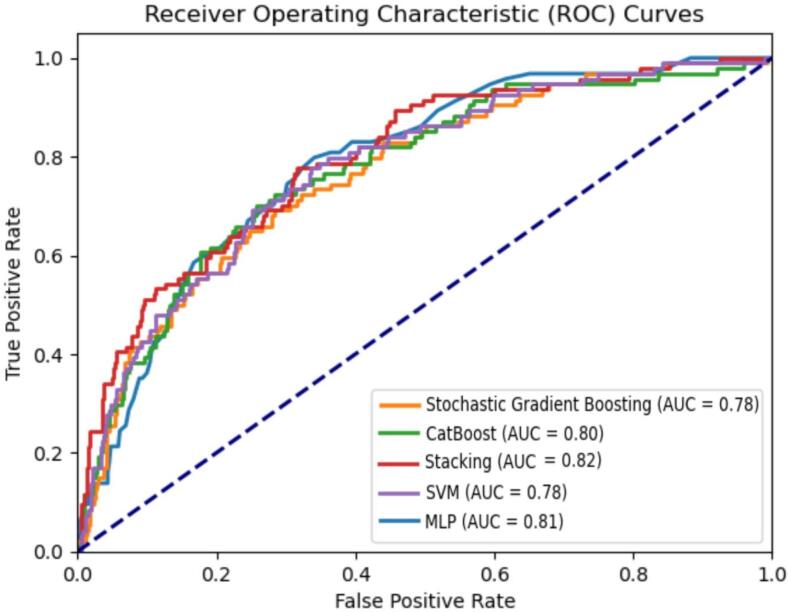
ROC curves comparing the performance of machine learning models. Mean ROC curve from five-fold cross-validation on the original dataset. ROC: receiver operating characteristic, AUC: area under the ROC curve.

We utilized the features to develop ML models. Each model was trained and evaluated using 5-fold cross-validation on the preprocessed dataset. The obtained results underscore that the accuracy of all models was 67 % or higher, in parallel with the minimum AUCROC of 0.75, as delineated in Table 3, Table 4. Stacking model outperformed other candidate classifiers in terms of AUCROC, achieving 0.801 and 0.778 for the train and test groups, retrospectively (Fig. 2). It also had the best sensitivities of 0.898 and 0.879 for train and test groups. In addition, Stochastic Gradient Boosting classifier depicted accuracies of 76.6 % and 73.4 % for train and test groups, as well as specificities of 0.763 and 0.753 for train and test groups, making it the best model in these metrics, retrospectively.

**Table 3:** Performance metrics for the machine learning classification models.

**Model**		**Accuracy**	**Sensitivity**	**Specificity**	**AUCROC**	**Balance accuracy**
**Stochastic Gradient Boosting**	**Train set**	0.766 (0.02)	0.787 (0.06)	0.763 (0.02)	0.798 (0.03)	0.727 (0.00)
	**Validation set**	0.759	0.782	0.762	0.781	0.725
**MLP**	**Train set**	0.709 (0.00)	0.895 (0.04)	0.689 (0.02)	0.797 (0.02)	0.689 (0.01)
	**Validation set**	0.701	0.896	0.675	0.788	0.676
**CatBoost**	**Train set**	0.704 (0.04)	0.871 (0.04)	0.674 (0.03)	0.779 (0.01)	0.683 (0.01)
	**Validation set**	0.693	0.868	0.673	0.761	0.679
**SVM**	**Train set**	0.723 (0.01)	0.870 (0.04)	0.707 (0.03)	0.792 (0.03)	0.702 (0.02)
	**Validation set**	0.717	0.861	0.698	0.784	0.687
**Stacking**	**Train set**	0.752 (0.04)	0.898 (0.05)	0.724 (0.04)	0.801 (0.05)	0.721 (0.00)
	**Validation set**	0.744	0.880	0.713	0.779	0.719

**Table 4:** Performance metrics for test group of the machine learning classification models.

**Model**	**Accuracy**	**Sensitivity**	**Specificity**	**AUCROC**	**Balance accuracy**
**Stochastic Gradient Boosting**	0.734	0.766	0.753	0.765	0.712
**MLP**	0.689	0.876	0.646	0.768	0.667
**CatBoost**	0.674	0.847	0.651	0.750	0.653
**SVM**	0.701	0.849	0.675	0.776	0.661
**Stacking**	0.729	0.879	0.703	0.778	0.708

When applied to the validation set, trends in the performance metrics were similar to the training set. Stochastic Gradient Boosting and SVM maintained high mean specificities above 69 %. MLP and CatBoost achieved the highest mean sensitivities of 89.6 % and 86.8 % respectively. The stacking ensemble performed well with mean AUC-ROC of 77.9 % and balanced accuracy of 71.9 %.

### Variable importance

**Fig. 3:**
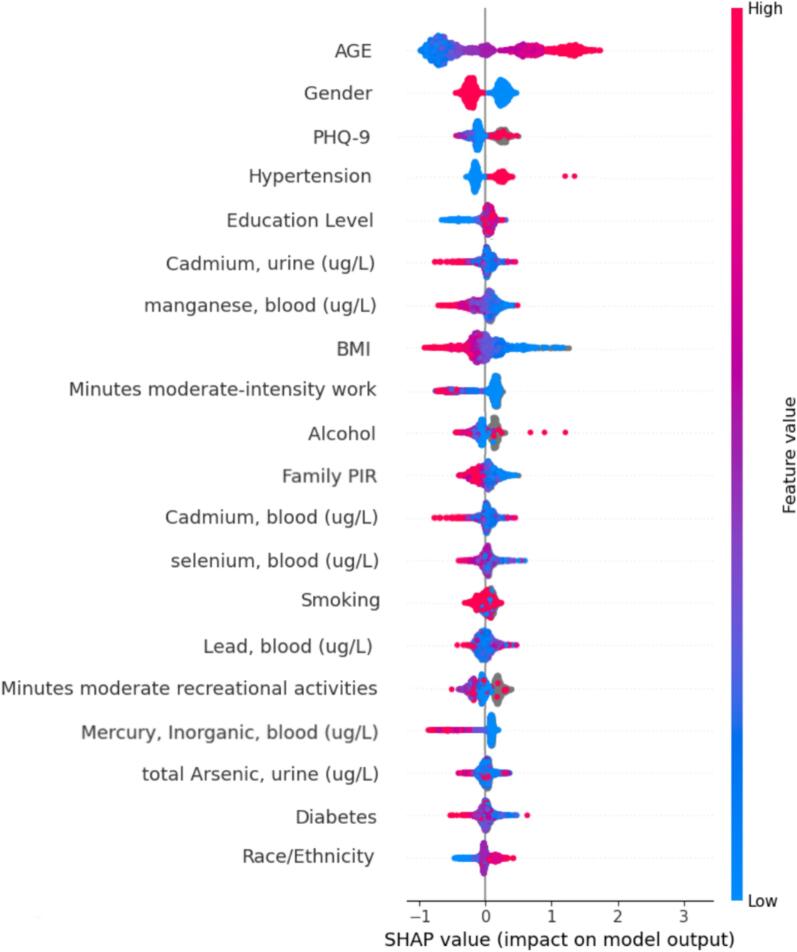
SHAP summary plot displaying feature importance rankings of the Stacking model. Each point represents a feature value, and different colors represent the final influence of the feature on the model output results, where red represents a larger value and blue represents a smaller value. PHQ: Patient Health Questionnaire, ug/L: micrograms per liter, BMI: body mass index, PIR: poverty to income ratio. (For interpretation of the references to color in this figure legend, the reader is referred to the web version of this article.)

In addition to the evaluation metrics, the feature importance that explains how the model reached its prediction was extracted from the Stacking model, demonstrating the ranking and relative magnitude of variables (Fig. 3). Age was found to be the most influential variable, followed by female gender. PHQ-9, hypertension, and educational level also had a strong impact on the prediction. Among HMs, the highest score was assigned to urine Cadmium and then blood Manganese. Supplementary figures S1-S4 present additional SHAP plots summarizing variable importance for the other evaluated models.

## Discussion

This study applied various ML algorithms to develop models for screening CI, utilizing a representative NHANES dataset, based on three groups of variables: demographics, CI risk factors, and levels of HMs’ exposure. Our work extends prior work by incorporating HMs’ exposure data into data-driven ML models designed to predict CI. According to Table 3, Table 4, Stochastic Gradient Boosting, MLP, CatBoost, and SVM achieved good accuracy scores on both the train and test sets, with values ranging from 0.704 to 0.766 on train sets and 0.674 to 0.734 on test sets. However, these models showed lower sensitivity compared to Stacking, with values between 0.766 and 0.879 on test sets. The Stacking classifier was identified as the best model due to the highest AUC (0.801) demonstrated in Table 3, showing the ability to handle the imbalanced nature of the data with the highest sensitivity (0.879) on the test set. Older female adults with underlying conditions like hypertension or depression were at the highest risk of CI. Urine cadmium and blood manganese were the most important determinants of the outcome among HMs.

Age, gender, race, BMI, education level, and underlying chronic diseases have been used to predict CI in previous ML studies (Li et al., 2023, Liu et al., 2023, Penfold et al., 2022, Wang et al., 2022). Although these studies achieved reasonable overall accuracy and AUC values, their reported sensitivities were quite low, with some models having sensitivities as low as 2 %. To address this, we explored techniques that have been successful in improving the sensitivity of models trained on imbalanced data in other fields, such as SMOTE, Cost-sensitive Learning which modifies learning algorithms to prioritize the minority class, and Feature Selection Techniques that focus on distinguishing features of the minority class (Abd Rani et al., 2013, Douzas et al., 2018). In addition to these techniques class weighting, assigning higher misclassification penalties for the minority class helped reduce the risk of achieving misleadingly high overall accuracy by failing to correctly identify any instances of the minority class. As CI can have serious health and quality of life implications, sensitivity may arguably be the most important performance metric to optimize, even if it comes at the cost of slightly lower specificity. Also, a low-cost, widely available diagnostic approach ensures we maximize the screening tool's sensitivity.

In this study, we have identified additional important factors that can be used as risk indicators to improve the performance of the models. Urine cadmium level, as our most valued HM, consistently was reported to have a negative correlation with cognitive function in cross-sectional analyses (Wang et al., 2022, Peng et al., 2020). The mechanism by which cadmium, as reflected in urine concentrations, affects cognitive function is not fully understood, but it is believed to involve neuronal apoptosis, disruption of neurotransmitter function, and oxidative stress in the brain, mainly through the induction of ROS (Samadhi and Irwanto, 2022). Overall, cadmium-induced apoptosis of cerebral cortical neurons occurs via mitochondrial calcium signaling, which is an intracellular ion that serves as a signaling mediator in many cellular processes, including proliferation, differentiation, and cell survival (Xu et al., 2011).

Another risk factor for CI in this study was blood manganese levels. Despite being an essential trace element with an important role in many biological processes and acting as an antioxidant in the body, chronically elevated levels of manganese have been found to accumulate in the basal ganglia region of the brain, similar to what is seen in Parkinson's disease. This accumulation disrupts normal neurotransmitter and antioxidant function, leading to oxidative stress and damage to neurons involved in motor control and cognition. Areas rich in dopamine and GABA neurons, such as the striatum and globus pallidus seem particularly susceptible. The resulting neuroinflammation and cell death may underlie the declines in cognitive abilities measured by tests like the CERAD and DSST (Barahona et al., 2022, Mogi, 2023).

The Stacking model outperformed other models with high sensitivity in predicting CI due to its ability to effectively integrate the outputs of base ML models. Through the stacking process, a *meta*-learner analyzed the predictions made by the individual base classifiers. By intelligently weighting and reconciling the strengths and weaknesses of each base model's predictions, the *meta*-learner could reduce overall errors and boost the ensemble's sensitivity. (Ghasemieh et al., 2023). This allowed the Stacking to take advantage of the unique strengths of different base models for pattern recognition, resulting in a more comprehensive evaluation of patient features. Rather than relying on simple voting schemes, the *meta*-learner in Stacking learning was specifically trained to minimize false negatives, an important quality for a model intended for clinical decision support (Kablan et al., 2023).

This study distinguishes itself from previous work by incorporating objective measurements of HM exposure data into ML models designed to predict CI. By analyzing complex interactions between multiple HM exposures and other risk factors, our ML algorithms could predict cognitive outcomes more accurately than traditional statistical methods. Integrating HM exposure data with demographics, clinical factors, and biomarkers into predictive models provides a more comprehensive assessment of CI risk. This novel approach has the potential to improve early detection and screening opportunities for at-risk individuals. We also utilized SHAP values to analyze our predictions and assess the influence of different covariates on predictions when applying complex ML models. To address the overfitting of our imbalanced data, we applied class-balancing techniques to preserve the sensitivity of the models. Additionally, a large sample size of 2933 participants drawn from the nationally representative NHANES dataset provided sufficient statistical power to train complex models.

However, some important limitations are worth mentioning. First, our dataset was cross-sectional, which limited our ability to determine the causal relationship between risk factors and CI. Second, we couldn’t account for individuals who had CI before being exposed to HM. Third, since our data was collected only in the United States, the differences across cultures might have impacted the prediction. Future prospective cohort studies are needed to validate predictive models and risk factors over time. Furthermore, validation of the ML models in other independent populations across countries will ensure the generalizability of the models.

### Ethics and consent to participate

This study was based on an analysis of deidentified, publicly available data from the National Health and Nutrition Examination Survey (NHANES) study. As the data analysis was performed on anonymous secondary data, ethics approval and participant consent were not required. NHANES is approved by the National Center for Health Statistics Research Ethics Review Board. Participants provided informed consent for clinical examination and use of genomic and biomarker data at the time of NHANES enrollment. Written informed consent was obtained from all NHANES participants prior to enrollment. No additional ethics approval was required for the current analysis.

## Author contributions

Study concept and design: AN; Acquisition of data: AN and FS; Statistical Analysis: AN, HMV, and MK, Analysis and interpretation of data: AN, MK, HMV, and SSN; Drafting of the manuscript: AN and FS, Critical revision of the manuscript for important intellectual content: AN, SSN, and FS; Study supervision: AN, HMV, and MK. All individuals listed as (co)-authors have met the authorship criteria, and nobody who qualifies for authorship is omitted from the list. The final manuscript was corrected and approved by all authors.

## CRediT authorship contribution statement

**Ali Nabavi:** Conceptualization, Methodology, Formal analysis, Investigation, Writing – original draft, Visualization. **Farimah Safari:** Resources, Data curation, Writing – review & editing, Supervision, Project administration. **Mohammad Kashkooli:** Software, Validation, Formal analysis, Writing – review & editing. **Sara Sadat Nabavizadeh:** Resources, Formal analysis. **Hossein Molavi Vardanjani:** Resources, Software, Writing – review & editing.

## Declaration of competing interest

The authors declare that they have no known competing financial interests or personal relationships that could have appeared to influence the work reported in this paper.

## Data Availability

The datasets analyzed during the current study are available in the National Health and Nutrition Examination Survey (NHANES) repository, [https://www.cdc.gov/nchs/nhanes/index.htm].
