# Supplementary material for: Early prediction of cognitive impairment in adults aged 20 years and older using machine learning and biomarkers of heavy metal exposure
Source: Curr Res Toxicol. 2024 Oct 18;7:100198. doi: 10.1016/j.crtox.2024.100198 (PMC11533558; doi:10.1016/j.crtox.2024.100198)

Figure S1. SHAP summary plot displaying feature importance rankings of the CatBoost model


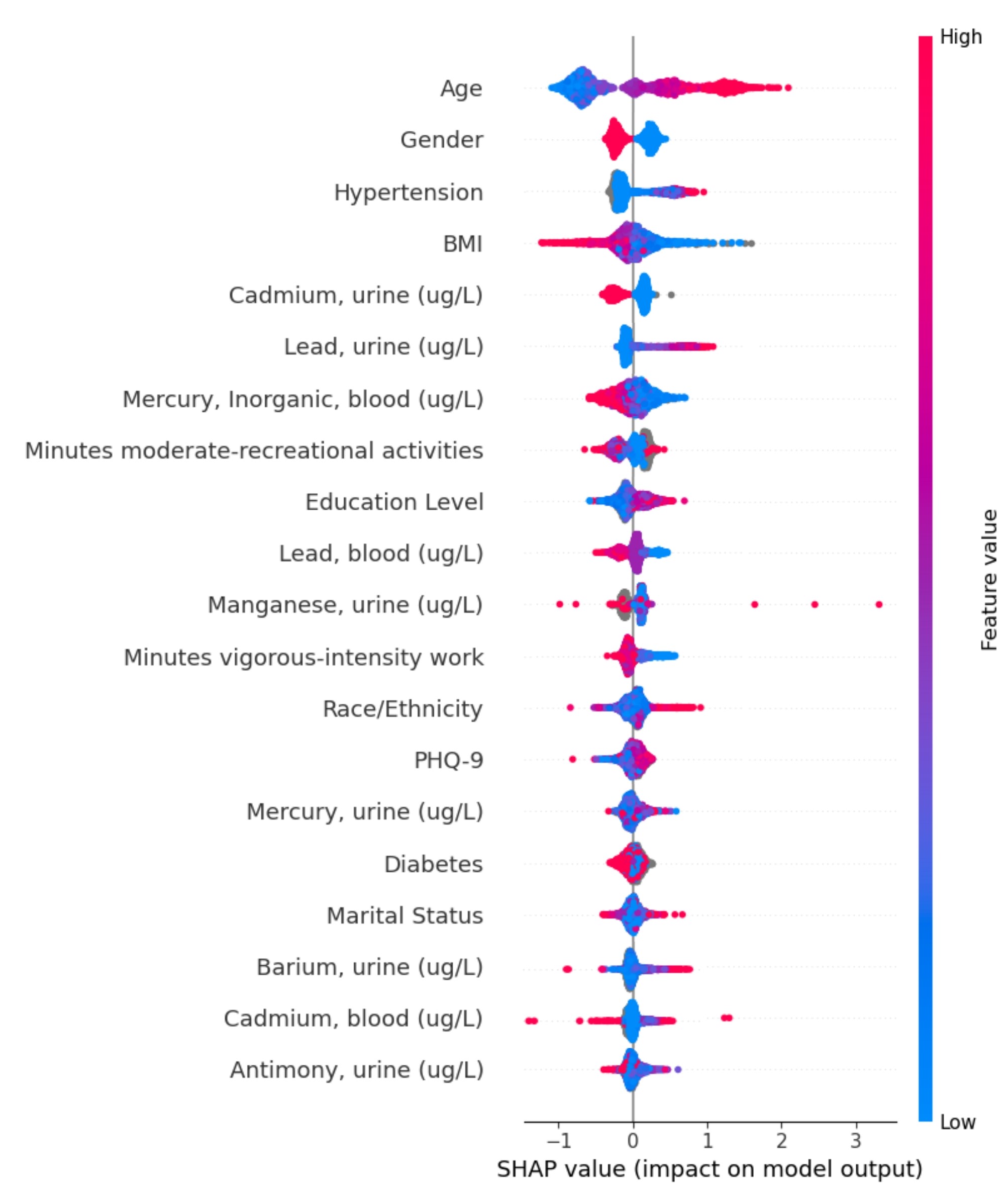


Figure S2. SHAP summary plot displaying feature importance rankings of the Stochastic Gradient Boosting model


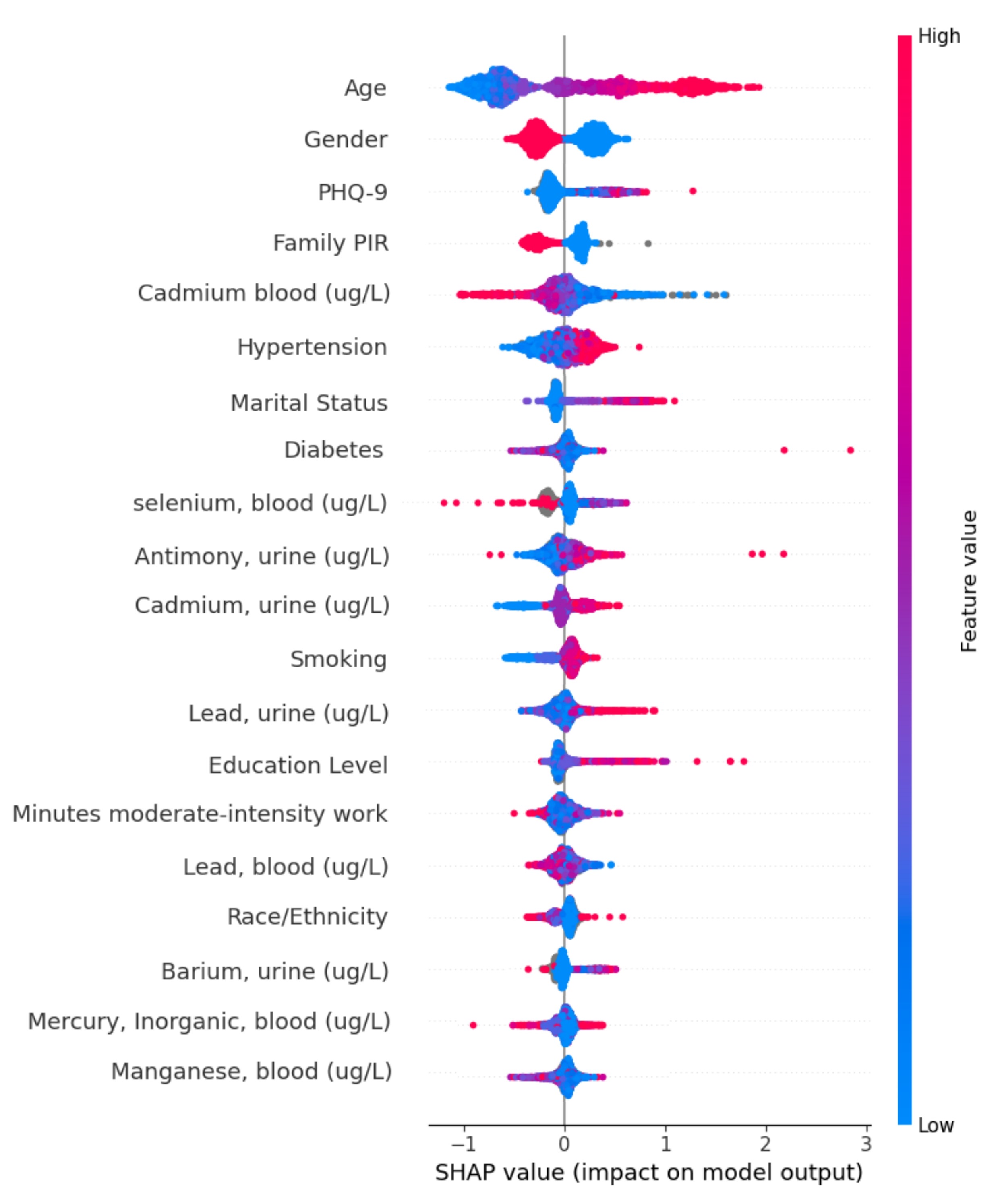


Figure S3. SHAP summary plot displaying feature importance rankings of the MLP model


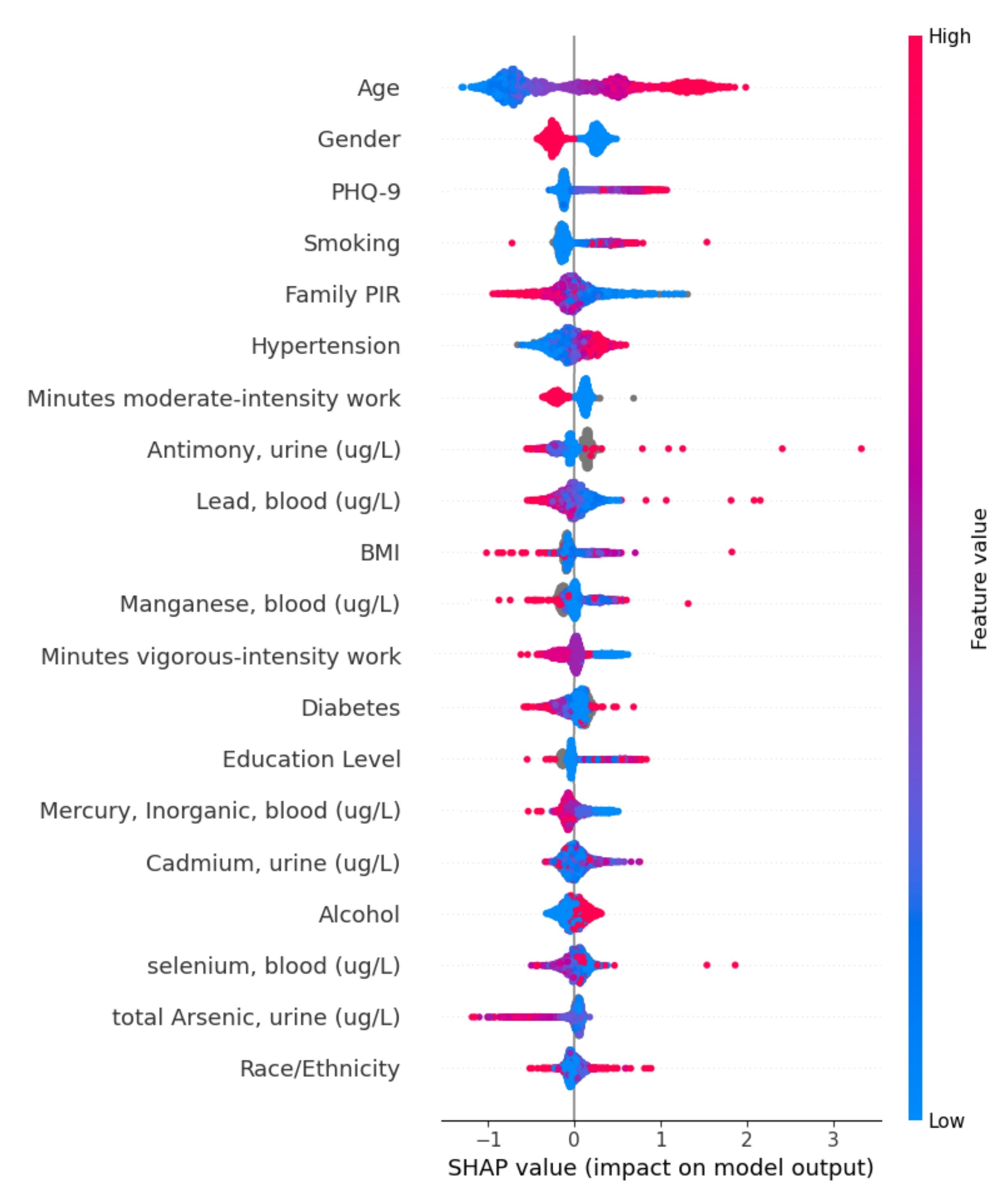


Figure S4. SHAP summary plot displaying feature importance rankings of the SVM model


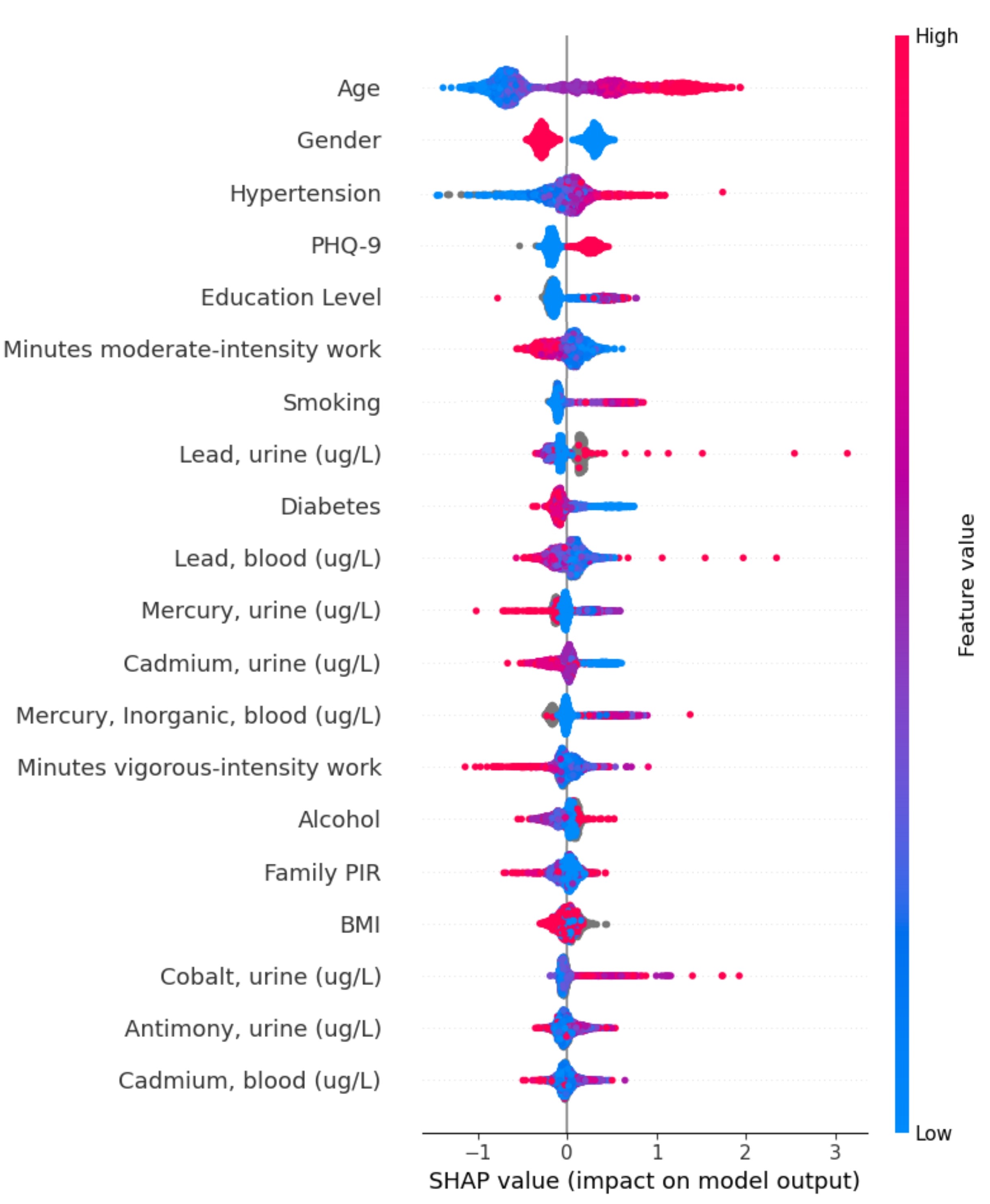

Supplement: Supplementary Data 1 [file mmc1.docx]
